# Number of MRI T1-hypointensity corrected by T2/FLAIR lesion volume indicates clinical severity in patients with multiple sclerosis

**DOI:** 10.1371/journal.pone.0231225

**Published:** 2020-04-03

**Authors:** Tetsuya Akaishi, Toshiyuki Takahashi, Kazuo Fujihara, Tatsuro Misu, Shunji Mugikura, Michiaki Abe, Tadashi Ishii, Masashi Aoki, Ichiro Nakashima

**Affiliations:** 1 Department of Neurology, Tohoku University Graduate School of Medicine, Sendai, Japan; 2 Department of Education and Support for Regional Medicine, Tohoku University Hospital, Sendai, Japan; 3 Department of Neurology, National Hospital Organization Yonezawa National Hospital, Yonezawa, Japan; 4 Department of Multiple Sclerosis Therapeutics, Fukushima Medical University School of Medicine, Fukushima, Japan; 5 Department of Diagnostic Radiology, Tohoku University Graduate School of Medicine, Sendai, Japan; 6 Department of Neurology, Tohoku Medical and Pharmaceutical University, Sendai, Japan; Kessler Foundation, UNITED STATES

## Abstract

**Introduction:**

Progressive brain atrophy, development of T1-hypointense areas, and T2-fluid-attenuated inversion recovery (FLAIR)-hyperintense lesion formation in multiple sclerosis (MS) are popular volumetric data that are often utilized as clinical outcomes. However, the exact clinical interpretation of these volumetric data has not yet been fully established.

**Methods:**

We enrolled 42 consecutive patients with MS who fulfilled the revised McDonald criteria of 2010. They were followed-up for more than 3 years from onset, and cross-sectional brain volumetry was performed. Patients with no brain lesions were excluded in advance from this study. For the brain volumetric data, we evaluated several parameters including age-adjusted gray-matter volume atrophy, age-adjusted white-matter volume atrophy, and T2-FLAIR lesion volume. The numbers of T1-hypointense and T2-FLAIR-hyperintense areas were also measured along the same timeline. The clinical data pertaining to disease duration, expanded disability status scale (EDSS), and MS severity score (MSSS) at the timing of volumetry were collected.

**Results:**

Among the 42 patients with MS and brain lesions, the number of T1-hypointensity (rho = 0.51, p<0.001), gray-matter atrophy (rho = 0.40, p<0.01) and white-matter atrophy (rho = 0.49, p<0.001) correlated with the EDSS. T1-hypointensity count divided by FLAIR lesion volume correlated with the MSSS (rho = 0.60, p<0.001). Meanwhile, counts or volumes of FLAIR-hyperintense lesions were associated only with the times of past relapses, and did not correlate with present neurological disability level or ongoing disease activity. These findings were consistent regardless of the presence of spinal cord lesions.

**Conclusion:**

Numbers of T1-hypointensities and brain atrophy equally indicated the current neurological disability in MS. The number of T1-hypointensities divided by FLAIR lesion volume represented the clinical severity. The size or number of FLAIR lesions reflected earlier relapses but was not a good indicator of neurological disability or clinical severity.

## Introduction

Multiple sclerosis (MS) is an autoimmune-related demyelinating disease involving the central nervous system [1–3]. Many risk factors associated with MS have been suggested, including Epstein-Barr virus infection [4, 5], vitamin D deficiency [6, 7], and smoking [8, 9], but the direct cause or exact pathological mechanism of MS is still unknown [10, 11]. Patients with typical MS usually show multiple demyelinating lesions present in the brain (i.e., juxta-cortical, peri-ventricular), optic nerves, or spinal cord [12–14]. Demyelinating lesions are present in T2-fluid-attenuated inversion recovery (FLAIR)-hyperintense areas, which sometimes contain T1-hypointense areas (i.e., T1-black hole) [15, 16]. The diagnostic criteria of MS generally comprise of the following two components: dissemination in time and dissemination in space [12, 17]. These components are evaluated based on the distribution of T2-FLAIR-hyperintense lesions (FL) and the presence of gadolinium enhancement [13, 18]. In addition to these demyelinating lesions, accelerated brain atrophy in gray matter (GM), as well as white matter (WM), is also known to occur in patients with MS [19–22].

With the establishment of brain volumetric methods, many volume-related variables (e.g., GM volume, WM volume, FL volume, T1-hypointense volume) are measured in MS patients. The primary imaging parameter for the diagnosis of MS is thought to be FL; however, its role as a surrogate parameter to reflect neurological disability or prognosis is uncertain [23]. Some previous reports indicated that brain atrophy or T1-hypointense areas (i.e., T1-black hole) may better reflect neurological disability—including cognitive impairment—and predict eventual neurological prognosis [24, 25]. The development of T1-hypointense lesions may result from localized hypo-perfusion and could be irreversible in cases of structural brain damage [26, 27]. Approximately 20% of contrast-enhancing lesions later evolve into persistent T1-hypointense areas [28]. Several reports have suggested that among MRI-related parameters, brain atrophy and especially gray-matter atrophy, has the best potential to predict clinical disability and cognitive impairment [29, 30]. However, it remains unknown whether brain atrophy or amount of T1-black hole would better reflect the concurrent neurological disability [21]. Therefore, the need for these data in routine clinical practice is still unmet [19, 31]. To maximize the clinical usability of brain volumetry, clarifying the clinical significance of each volumetric variable is essential. In this study, we enrolled a feasible number of patients with MS and brain lesions. Each patient underwent brain volumetry and was followed-up for more than 3 years. These efforts comprised an attempt to clarify the clinical utility of each volumetric data for the management of patients with MS.

## Methods

### Enrollment

This study enrolled 42 consecutive patients with MS (14 males and 28 females) who were treated in a single university hospital in Japan, and followed-up for more than 3 years until 2017. Each patient underwent brain volumetry once between 2016 and 2017. All patients fulfilled the revised McDonald criteria of 2010. Because this study tried to clarify the clinical significance of brain volumetry, those with no brain lesions (n = 15) were excluded in advance from this study. Patients with a radiologically isolated syndrome (n = 3), who were incidentally found to have T2-hyperintense brain lesions, were also excluded in advance. All the enrolled patients were confirmed to be negative for serum anti-aquaporin 4 (AQP4) antibody and anti-myelin oligodendrocyte glycoprotein (MOG) antibody in advance by utilizing a live cell-based assay (CBA) method [32, 33].

### Clinical variables

The demographic data of the enrolled patients, sex, onset age, and disease duration at the time of brain volumetry were collected. Concerning the data related to disease severity and activity, the expanded disability status scale (EDSS) [34], MS severity score (MSSS) [35], and number of past clinical relapses at the time of volumetry were collected. MSSS allows cross-sectional comparison of clinical severity between patients with different disease durations. The score is assigned to each patient according to the present EDSS and disease duration. To adjust for the effect of the site of the responsible lesions (*i*.*e*., brain, optic nerves, or spinal cord), the site mainly contributing to the EDSS level in each patient was evaluated.

### Brain magnetic resonance imaging data

MR imaging was performed on a 3 Tesla whole-body clinical system (IngeniaCX or Achieva dStream, Philips Healthcare, Best, The Netherlands) using a 32-channel anterior and posterior phased-array coil. The protocol contained two 3D sequences: a fat-saturated 3D FLAIR (TR: 4800 ms, TE: 268 ms, TI: 1650 ms, 240 × 240 mm^2^ field of view (FOV), 180 sagittal slices, 1.0 × 1.0 × 1.0 mm^3^ voxel resolution) and a 3D T1-weighted fast field echo (FFE) sequence (TR: 10 ms, TE: 5.7 ms, FA: 8°, 240 × 240 mm^2^ FOV, 180 sagittal slices, 1.0 × 1.0 × 1.0 mm^3^ voxel resolution). Brain volumetry was determined by uploading the DICOM data (3-D FLAIR and 3-D T1 MP-RAGE images) to the Icometrix website (Leuven, Belgium; Chicago, USA), as reported previously [21, 36]. The method utilized both the T1-weighted and the FLAIR images in an iterative process to obtain tissue (i.e. WM, GM, cerebrospinal fluid) and lesion segmentations. A lesion-filled T1-weighted image was created by replacing lesion voxels with values matching the WM tissue intensities. Thus, in the final lesion-filled T1-weighted image segmentation, FL lesions were included in the WM segment. Brain GM volume, WM volume, and FL volume were provided in the analysis report. Age-adjusted GM atrophy and age-adjusted WM atrophy were cross-sectionally estimated for each patient by subtracting the age-adjusted mean of volume in normal controls, which was provided by Icometrix, from the measured volume in each patient. In addition to these data, the number of T1-hypointense areas (T1-count) and FL (FL-count) were simultaneously counted in each patient. The volumes of T1-hypointense areas were not included in the volumetric data analysis in this study. As described later in the results section, the T1-count and FL-volume showed a moderate-to-strong positive correlation, but only T1-count showed a significant correlation with EDSS or MSSS. Thus, we also calculated the ratio of T1-count and FL-volume (T1-count/FL-volume), and checked the correlations between the ratio and the studied clinical variables.

### Statistical analyses

The correlation between each volumetric and clinical data parameter was evaluated by the Spearman’s correlation coefficient (rho) because of the non-normal distribution of the evaluated variables, followed by a test of no correlation. The multiple regression analysis comparing the contribution to the MSSS level was performed using the present age, GM atrophy, and T1-count/FL-volume as the explanatory variables. Because multiple comparisons were performed, p-values less than 0.01 were considered statistically significant. Statistical analyses were conducted using the SPSS Statistics Base 22 software (IBM, Armonk, NY, USA) and MATLAB R2015a (MathWorks, Natick, MA, USA).

### Ethics statement

This study was approved by the Institutional Review Board of Tohoku University Graduate School of Medicine (THK 2010589). Written informed consent was obtained from all the participants enrolled in this study. The study processes were performed according to standard regulations.

## Results

### Patients’ backgrounds

The mean ± standard deviation (SD) of onset age in the enrolled 42 patients was 26.7 ± 8.3 years, and the mean disease duration at the time of brain volumetry was 12.4 ± 7.5 years. Concerning the disease modifying therapies during brain volumetry, 4 were untreated, 28 were treated with fingolimod, 8 with interferon-beta injection, 2 with dimethyl fumarate, 1 with glatiramer acetate, and 1 with natalizumab. The clinical types of MS at the timing of volumetry were as follows: 30 had relapsing-remitting MS, 10 had secondary-progressive MS, and 2 had primary-progressive MS.

The median and interquartile range (IQR, 25–75 percentiles) of EDSS during brain volumetry were 2.0 (1.0–3.5). The mean ± SD of MSSS during brain volumetry was 3.37 ± 2.60. All EDSS data in this study were evaluated in the chronic phase, at least 3 months after the last clinical relapse. Details of each enrolled patient are summarized in **S1 Table**.

### Measured volumetric data

Mean ± SD of the age-adjusted GM atrophy was 106 ± 51 [cc], and that of age-adjusted WM atrophy was 44 ± 43 [cc]. Median (IQR) of FL-volume was 15.0 (9.3–25.0) [cc], the median T1-count was 5 (2–12), and median FL-count was 18 (12–32). Mean ± SD of the T1-count/FL-volume was 0.42 ± 0.30 [/cc], and the mean T1-count/FL-count was 0.32 ± 0.24.

### Correlations between magnetic resonance imaging-based variables

**Fig 1:**
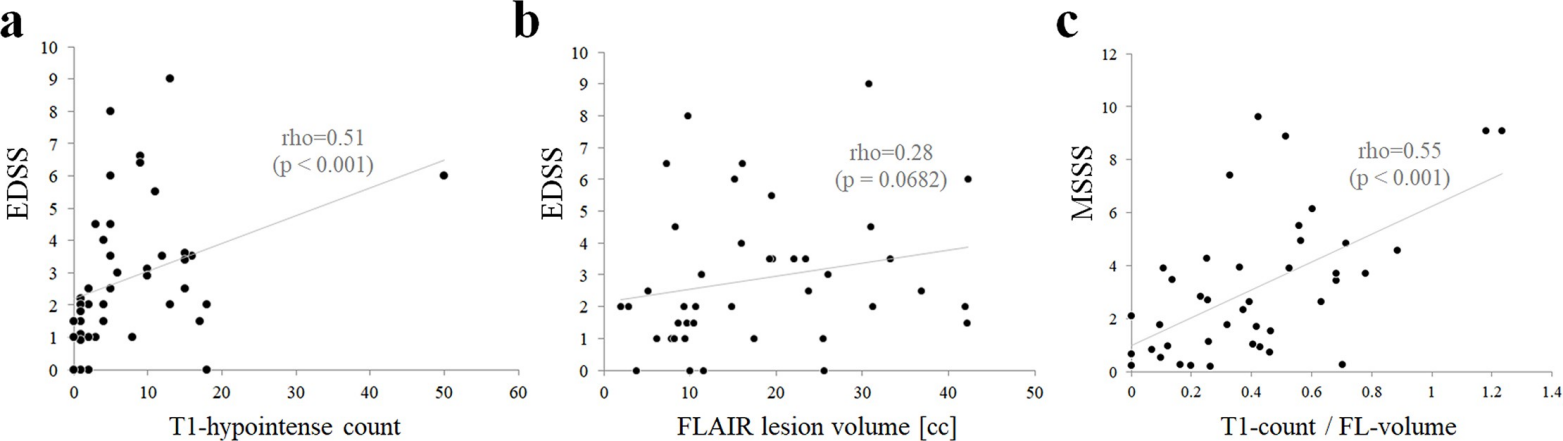
Scatter plots of MRI-derived variables, and neurological disability. (a) Scatter plot of the number of T1-hypointense areas and EDSS. (b) Scatter plot of FL volume and EDSS. (c) Scatter plot of the ratio of T1-hypointense count/FL-volume and MSSS. The grey linear lines are the approximate straight lines. Abbreviations: FL, FLAIR-hyperintense lesion; MSSS, multiple sclerosis severity scores.

A correlation matrix between the raw magnetic resonance imaging (MRI)-derived variables is shown in **Table 1**. GM atrophy and WM atrophy showed a weak positive correlation. Both FL-volume and T1-count showed moderate positive correlations with WM atrophy; however, FL-volume and T1-count showed a strong positive correlation. T1-count showed significant correlations with the levels of EDSS (**Fig 1A**) and MSSS, but it did not correlate with the ARR. Meanwhile, FL-volume had a significant correlation with ARR but not with EDSS (**Fig 1B**) or MSSS.

**Table 1:** A correlation matrix with volumetric data in the enrolled 42 patients.

	GM atrophy	WM atrophy	FL-volume	T1-count	FL-count
GM atrophy	-	0.34 (p = 0.0273)	0.47* (p = 0.0019)	0.38 (p = 0.0131)	0.09 (p = 0.59)
WM atrophy	-	-	0.43* (p = 0.0046)	0.56** (p<0.001)	0.32 (p = 0.0389)
FL-volume	-	-	-	0.66** (p<0.001)	0.71** (p<0.001)
T1-count	-	-	-	-	0.63** (p<0.001)
*Clinical data during brain volumetry*					
Sex (female)	-0.43* (p = 0.0046)	-0.15 (p = 0.34)	-0.12 (p = 0.45)	0.07 (p = 0.64)	0.08 (p = 0.61)
Onset age	0.10 (p = 0.55)	0.06 (p = 0.70)	-0.15 (p = 0.33)	-0.08 (p = 0.62)	-0.23 (p = 0.14)
Duration	0.52 (p<0.001)	0.19 (p = 0.22)	0.43* (p = 0.0043)	0.19 (p = 0.21)	0.12 (p = 0.45)
EDSS	0.40* (p = 0.0083)	0.49** (p<0.001)	0.28 (p = 0.0682)	0.51** (p<0.001)	0.29 (p = 0.0652)
MSSS	0.11 (p = 0.55)	0.33 (p = 0.0310)	0.02 (p = 0.90)	0.42* (p = 0.0053)	0.18 (p = 0.24)
Total relapses	0.11 (p = 0.55)	0.15 (p = 0.40)	0.57** (p<0.001)	0.37 (p = 0.0333)	0.54* (p = 0.0013)

The correlation coefficients displayed are Spearman’s rho, together with the p-values by the test of no correlation. GM: gray matter, WM: white matter, FL: T2-FLAIR-hyperintense lesion, T1-couunt: number of T1-hypointense areas, EDSS: expanded disability status scale, MSSS: multiple sclerosis severity score.

* p<0.01

** p<0.001.

### T1-hypointense count adjusted by FLAIR lesion volume

Because the T1-count showed a strong positive correlation with FL-volume, we calculated the ratio of T1-count to FL-volume (T1-count/FL-volume). The correlation matrix employing this T1-count/FL-volume is shown in **Table 2**, together with the matrix for other four types of MRI-derived data divided by FL-count or -volume. As can be seen, T1-count/FL-volume more significantly reflects the level of MSSS than the other duration-adjusted MRI data. A scatter plot with these two variables is shown in **Fig 1C**. The present age, onset age, or disease duration did not affect the correlation between T1-count/FL-volume and MSSS.

**Table 2:** Correlation matrix between volumetry-derived variables and disease severity.

	GM atrophy / FL-volume	WM atrophy / FL-volume	FL-volume / FL-count	T1-count / FL-volume	T1-count / FL-count
Sex (female)	-0.19 (p = 0.22)	-0.07 (p = 0.67)	-0.19 (p = 0.23)	0.19 (p = 0.22)	-0.02 (p = 0.92)
Onset age	0.13 (p = 0.42)	0.12 (p = 0.44)	0.08 (p = 0.60)	0.03 (p = 0.84)	0.15 (p = 0.36)
Duration	-0.05 (p = 0.73)	-0.03 (p = 0.84)	0.41* (p = 0.0075)	-0.12 (p = 0.46)	0.28 (p = 0.0739)
EDSS	0.02 (p = 0.88)	0.20 (p = 0.21)	-0.01 (p = 0.96)	0.44* (p = 0.0032)	0.45* (p = 0.0029)
MSSS	0.09 (p = 0.59)	0.20 (p = 0.20)	-0.21 (p = 0.20)	0.55** (p<0.001)	0.39 (p = 0.0117)
Total relapses	-0.50* (p = 0.0025)	-0.24 (p = 0.16)	0.01 (p = 0.93)	0.16 (p = 0.36)	0.18 (p = 0.32)

Shown correlation coefficients are Spearman’s rho, together with the p-values by the test of no correlation. GM: gray matter, WM: white matter, FL: T2-FLAIR-hyperintense lesion, T1 count: number of T1-hypointense area, EDSS: expanded disability status scale, MSSS: multiple sclerosis severity score.

* p<0.01

** p<0.001.

### Multiple regression analysis

Next, we compared the significance of age-adjusted GM atrophy and T1-count/FL-volume for the level of MSSS by performing a multiple regression analysis. Here, only the ratio of T1-count/FL-volume (F(3,38) = 24.46, p<0.0001) showed a statistically significant contribution to MSSS. In contrast, GM atrophy (F(3,38) = 3.03, p = 0.0896) and present age (F(3,38) = 0.17, p = 0.68) did not significantly contribute to MSSS.

### Presence of spinal cord lesions

Finally, we analyzed the correlation coefficients between the MRI-derived variables and clinical variables only among patients who had no spinal cord lesions (n = 24). The achieved correlation matrix is shown in **Table 3**. The results obtained in the previous sections were true even when analyzed among the patients who had no spinal cord lesions.

**Table 3:** Correlation matrix among the patients without spinal cord lesions (n = 24).

	EDSS	MSSS	Total relapses
GM atrophy	0.53* (p = 0.0078)	0.15 (p = 0.48)	0.11 (p = 0.65)
WM atrophy	0.62* (p = 0.0011)	0.43 (p = 0.0350)	0.26 (p = 0.27)
FL-volume	0.41 (p = 0.0489)	0.01 (p = 0.96)	0.65* (p = 0.0018)
T1-count	0.53* (p = 0.0067)	0.44 (p = 0.0276)	0.16 (p = 0.50)
FL-count	0.37 (p = 0.0875)	0.23 (p = 0.31)	0.64* (p = 0.0046)
GM atrophy / FL-volume	-0.10 (p = 0.65)	0.00 (p = 0.99)	-0.60* (p = 0.0054)
WM atrophy / FL-volume	0.06 (p = 0.80)	0.15 (p = 0.47)	-0.30 (p = 0.20)
FL-volume / FL-count	0.05 (p = 0.82)	-0.28 (p = 0.21)	-0.16 (p = 0.53)
T1-count / FL-volume	0.50 (p = 0.0166)	0.66** (p<0.001)	0.01 (p = 0.96)
T1-count / FL-count	0.48 (p = 0.0228)	0.42 (p = 0.0488)	0.01 (p = 0.97)

Shown correlation coefficients are Spearman’s rho, together with the p-values by the test of no correlation. GM: gray matter, WM: white matter, FL: T2-FLAIR-hyperintense lesion, T1 count: number of T1-hypointense area, EDSS: expanded disability status scale, MSSS: multiple sclerosis severity score.

* p<0.01

** p<0.001.

## Discussion

Many MRI imaging-derived parameters have been evaluated previously as indicators or predictors of neurological disability in patients with MS. Previous studies demonstrated that accelerated GM atrophy in patients with MS drives neurological disability progression in this population [29]. Other reports have suggested that the accumulation of T2-FLAIR-hyperintense WM lesions could reflect the cognitive dysfunction in MS, although the results differed among the various studies [37]. Other reports suggested that T1-hypointense areas are more important than T2-FLAIR-hyperintense lesions for reflecting the ongoing neurological damage and determining the eventual neurological prognosis. Although the clinical importance of each of these imaging parameters has been separately shown, which of the parameters should be the most clinically important, and what exactly each of the parameters clinically indicates in the management of patients with MS has not been fully elucidated. In this study, we demonstrated the importance of the amount of T1-hypointense areas, together with the brain atrophy, as a parameter of concurrent neurological disability. When the count of T1-hypointense areas was divided by FL-volume, it well represented the clinical severity in patients with MS. Meanwhile, the volume of T2/FLAIR-hyperintense lesions did not show a significant correlation with current neurological disability or the ongoing disease activity.

Another notable finding of this study was that a simple T1-count could efficiently reflect the clinical severity and disease activity. Because T1-count can be manually counted without specific volumetric software, this simple information would be quite useful in routine clinical practice to estimate the ongoing disease activity in each MS patient. If the brain volumetric data can be achieved concurrently, these data would surely add more clinically useful information. In some previous reports, in addition to the lesion load, the location of T2/FLAIR lesions (e.g. corticospinal tract, superior longitudinal fasciculus, inferior fronto-occipital fasciculus) was also shown to impact the neurological disability level and the progression rate [38, 39]. Considering both the amount of T1-hypointense areas within T2/FLAIR lesions and the location of them may further facilitate the clinical utility of MRI-derived parameters.

There were some limitations to this study. The major limitation was that we did not evaluate the volume of T1-hypointense areas. Since this study was strongly based on MRI measures of T1-hypointense lesions, this limitation does not allow us to answer which of brain atrophy and development of T1-black hole would better reflect the concurrent neurological disability and clinical severity. Further research that measures the volume of T1-hypointense areas is needed to conclude this point. Another limitation is was that our study cohort was relatively small, and consisted entirely of Asian patients. Studies with larger sample sizes, which include patients of other ethnicities, are needed to confirm whether our conclusions can be generalized to Caucasian and African-American patients with MS. The final limitation was that we did not assess cognitive impairment in this study. Thus, which of the T1-hypointense area and GM atrophy may more efficiently reflect the cognitive impairment is still unknown. As a future perspective, comprehensive comparisons between the above-described imaging parameters and clinical variables other than EDSS (e.g., cognitive impairment, fatigue) should be targets of future clinical research.

## Conclusions

The number of T1-hypointense areas, corrected for T2-FLAIR-hyperintense lesion volume, reflects the disease severity and activity in patients with MS. Further research is needed to determine the importance of regular follow ups of the number and volumes of T1-hypointense areas, along with FL-volume, to evaluate treatment efficacy or to estimate the eventual neurological prognosis in patients with MS.

## Supporting information

S1 Table Clinical and imaging variables of the enrolled patients with MS. (XLSX)

## Abbreviations

ARR: annual relapse rate
EDSS: expanded disability status scale
FL: T2-FLAIR-hyperintense lesions
GM: gray matter
MS: multiple sclerosis
MSSS: MS severity score
PPMS: primary-progressive MS
RRMS: relapsing-remitting MS
SPMS: secondary-progressive MS
WM: white matter
